# Effects of *Bi*lberry and *O*at intake on lipids, inflammation and exercise capacity after *A*cute *M*yocardial *I*nfarction (BIOAMI): study protocol for a randomized, double-blind, placebo-controlled trial

**DOI:** 10.1186/s13063-021-05287-5

**Published:** 2021-05-10

**Authors:** Cecilia Bergh, Rikard Landberg, Kristina Andersson, Lovisa Heyman-Lindén, Ana Rascón, Anders Magnuson, Payam Khalili, Amra Kåregren, Johan Nilsson, Carlo Pirazzi, David Erlinge, Ole Fröbert

**Affiliations:** 1 Clinical Epidemiology and Biostatistics, School of Medical Sciences, Örebro University, 701 85 Örebro, Sweden; 2 Department of Biology and Biological Engineering, Food and Nutrition Science, Chalmers University of Technology, Gothenburg, Sweden; 3 Department of Public Health and Clinical Medicine, Umeå University, Umeå, Sweden; 4 Department of Experimental Medical Science, Lund University, Lund, Sweden; 5 Glucanova AB, Lund, Sweden; 6 Molecular Nutrition, Department of Experimental Medical Science, Lund University, Lund, Sweden; 7 Berry Lab AB, Lund, Sweden; 8 Department of Food Technology, Engineering and Nutrition, Lund University, Lund, Sweden; 9 Department of Cardiology and Acute Internal Medicine, Central Hospital, Karlstad, Sweden; 10 Department of Medicine, Hospital Region Västmanland, Västerås, Sweden; 11 Department of Cardiology, Sahlgrenska University Hospital, Gothenburg, Sweden; 12 Department of Cardiology, Clinical Sciences, Lund University, Lund, Sweden; 13 Department of Cardiology, Faculty of Medicine and Health, Örebro University, Örebro, Sweden

**Keywords:** Anthocyanin, Anthocyanin-derived phenolic acid metabolites, Bilberry, Cholesterol, Diet therapy, Exercise test, Inflammation, Myocardial infarction

## Abstract

**Background:**

Bilberries from Sweden, rich in polyphenols, have shown cholesterol-lowering effects in small studies, and the cholesterol-lowering properties of oats, with abundant beta-glucans and potentially bioactive phytochemicals, are well established. Both may provide cardiometabolic benefits following acute myocardial infarction (AMI), but large studies of adequate statistical power and appropriate duration are needed to confirm clinically relevant treatment effects. No previous study has evaluated the potential additive or synergistic effects of bilberry combined with oats on cardiometabolic risk factors. Our primary objective is to assess cardioprotective effects of diet supplementation with dried bilberry or with bioprocessed oat bran, with a secondary explorative objective of assessing their combination, compared with a neutral isocaloric reference supplement, initiated within 5 days following percutaneous coronary intervention (PCI) for AMI.

**Methods:**

The effects of *Bi*lberry and *O*at intake on lipids, inflammation and exercise capacity after *A*cute *M*yocardial *I*nfarction (BIOAMI) trial is a double-blind, randomized, placebo-controlled clinical trial. A total of 900 patients will be randomized post-PCI to one of four dietary intervention arms. After randomization, subjects will receive beverages with bilberry powder (active), beverages with high-fiber bioprocessed oat bran (active), beverages with bilberry and oats combined (active), or reference beverages containing no active bilberry or active oats, for consumption twice daily during a 3-month intervention. The primary endpoint is the difference in LDL cholesterol change between the intervention groups after 3 months. The major secondary endpoint is exercise capacity at 3 months. Other secondary endpoints include plasma concentrations of biochemical markers of inflammation, metabolomics, and gut microbiota composition after 3 months.

**Discussion:**

Controlling hyperlipidemia and inflammation is critical to preventing new cardiovascular events, but novel pharmacological treatments for these conditions are expensive and associated with negative side effects. If bilberry and/or oat, in addition to standard medical therapy, can lower LDL cholesterol and inflammation more than standard therapy alone, this could be a cost-effective and safe dietary strategy for secondary prevention after AMI.

**Trial registration:**

ClinicalTrials.gov NCT03620266. Registered on August 8, 2018.

**Supplementary Information:**

The online version contains supplementary material available at 10.1186/s13063-021-05287-5.

## Background

Cardiovascular disease (CVD) is a leading cause of death and disability globally. Dyslipidemia is a major modifiable risk factor for the development of atherosclerotic plaques [1]. Systemic inflammation also plays a central role in the atherosclerotic process from initiation through progression and rupture of plaques [2, 3]. Observational studies suggest that the risk of acute myocardial infarction (AMI) in persons with hyperlipidemia is three times that of the population with normal lipid status. A reduction in serum cholesterol is strongly associated with a reduction in CVD risk [4, 5]. Secondary prevention after AMI has improved in recent decades, but readmissions and death following AMI remain important challenges. Although new lipid-lowering medications are effective in reducing low-density lipoprotein (LDL) cholesterol levels, clinical guidelines advocate addition of non-statin agents [6], but their cost/efficacy is debatable even in high-risk patients [7].

From a public health perspective, lifestyle modification, including dietary changes, is considered a first step in controlling and treating CVD risk factors [8]. However, patients at highest risk of premature death, AMI, and re-hospitalization are those with known CVD [9]. Consequently, international guidelines recommend secondary prevention strategies to reduce subsequent cardiovascular risk, such as evidence-based pharmacological therapy and adherence to diet and physical activity regimens [10]. Studies have shown positive effects on cardiovascular risk factors of a Nordic diet, in which berries and oats are important elements [11, 12]. However, food supplement clinical trials that include subjects with overt disease are scarce, and there is a need for well-planned and well-executed controlled intervention studies of sufficient size and duration to provide a firm evidence base for dietary guidance in both healthy individuals and those suffering from heart disease [13–15].

A meta-analysis of more than 50 clinical trials concluded that oats can lower LDL cholesterol by 5–8% [16], and subsequent data suggest other anti-atherogenic properties of oats, including reducing inflammation and oxidative stress [17]. Recent studies indicate that bilberries from Sweden, rich in polyphenols and anthocyanins, may have direct or indirect positive effects on cardiovascular risk factors [13, 18, 19]. It has been suggested that consumption of the anthocyanins present in bilberries may reduce risk of death from CVD [20–22], have beneficial effects on platelet function, increase high-density lipoprotein (HDL) cholesterol [23, 24], and improve exercise tolerance [25, 26]. The mechanisms of action of berries are likely to be different from those of oats, potentially providing additive or synergistic beneficial effects. We recently conducted a small open-label randomized clinical trial of bilberry supplementation in patients post-AMI receiving high-dose statin treatment and found significant beneficial effects on lipid profile and exercise capacity in patients receiving bilberry supplementation [27]. We are proceeding to conduct a large, randomized, placebo-controlled clinical trial to assess clinical impact.

## Methods

### Study design, hypotheses, and primary and secondary endpoints

The effects of *Bi*lberry and *O*at intake on lipids, inflammation and exercise capacity after *A*cute *M*yocardial *I*nfarction (BIOAMI) trial is a prospective, randomized, double-blind, placebo-controlled multicenter trial in patients post-AMI. The primary objective is to assess differences in LDL cholesterol change among treatments after 3 months of a diet supplemented with a beverage containing either dried bilberry or bioprocessed oat bran compared with a neutral isocaloric reference drink, initiated within 5 days following percutaneous coronary intervention (PCI) for AMI. A secondary objective is to assess the combination of bilberries and oats compared with the reference drink. We hypothesize that standard medical therapy supplemented with either dried bilberries or bioprocessed oat bran post-AMI will show a more beneficial effect on LDL cholesterol than medical therapy alone after 3 months. Additional secondary objectives are to determine effects on exercise capacity, biomarkers of inflammation and other biochemical markers, resting heart rate, blood pressure, and left ventricular systolic function (Table 1). We will also employ untargeted metabolomics to exploratively assess alterations in endogenous and exposome-related metabolites and effects on gut microbiota composition and activity. These exploratory analyses will allow us to investigate the extent to which gut microbiota composition and activity differs between responders and non-responders to the interventions. At 1-year following randomization, we will exploratively assess clinical endpoints in all randomized patients using data from the Swedish Coronary Angiography and Angioplasty Registry (SCAAR) with respect to death, new AMI, and new unplanned revascularization.

**Table 1:** Primary and secondary endpoints

Primary endpoint • LDL-C cholesterol Major secondary endpoint • Symptom-limited bicycle ergometer test (Watts and estimated VO_2_max) Other secondary endpoints • Dynamic unilateral heel-lift and unilateral shoulder-flexion tests (*n*) • Self-reported activity level (levels 1–6, days/week) • Fasting lipid levels (TC, HDL-C, TGA, small dense LDL-C, apo A, apo B, Lp(a), oxidized LDL) • Fasting insulin, c-peptide, creatinine, glucose, cystatin C • HbA1c, hs-CRP, NT-proBNP, troponin-I, IL-6 • Resting heart rate and blood pressure • Left ventricular systolic function • Untargeted plasma metabolome • Gut microbiota composition	

### Study population and patient selection

Subjects in this multicenter study will be recruited from among patients referred to Sahlgrenska University Hospital Gothenburg, Umeå University Hospital, Lund University Hospital, Västerås general hospital, Karlstad general hospital, and Örebro University Hospital for coronary angiography/PCI due to ST-segment elevation myocardial infarction (STEMI) or non-ST-segment elevation myocardial infarction (NSTEMI). STEMI is defined as chest pain suggestive of myocardial ischemia for at least 30 min prior to hospital admission with time from onset of symptoms less than 24 h, an ECG with new ST-segment elevation in two or more contiguous leads of ≥ 0.2 mV in leads V2–V3 and/or ≥ 0.1 mV in other leads, or a probable new-onset left bundle branch block. NSTEMI is defined as a combination of onset of symptoms such as central chest pain or aggravated angina pectoris, with or without ECG changes, with ST-segment depression or an inverted T-wave and rise and/or fall of troponin-T or troponin-I above the established margin of an AMI. Study inclusion and exclusion criteria are defined in Table 2.

**Table 2:** Inclusion and exclusion criteria

Inclusion criteria • STEMI or NSTEMI • Completed coronary angiography/PCI • Male and female subjects ≥ 18 years • Allocated to atorvastatin at a daily dose of 80 mg • Written informed consent Exclusion criteria • Emergency coronary artery bypass grafting • < 18 years of age • LDL cholesterol < 2.0 mmol/L • Daily intake or the intent to initiate daily intake of bilberry in any form or daily intake of > 15 g of oatmeal or equivalent • Previous randomization in the BIOAMI trial • Inability to provide informed consent	

### Randomization

**Fig. 1:**
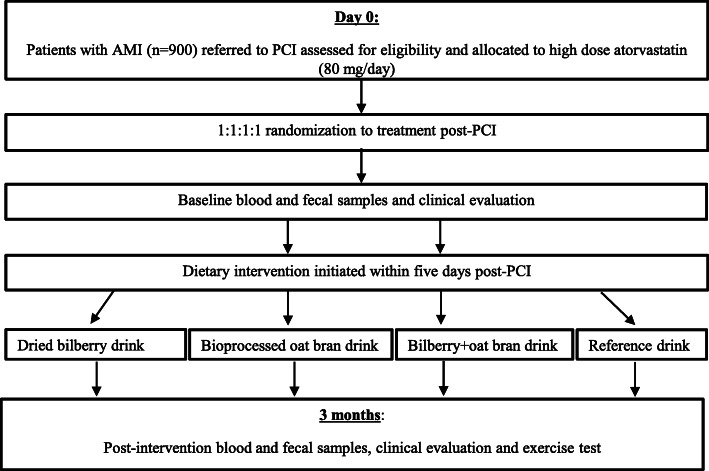
Flowchart of study design. AMI, acute myocardial infarction; PCI, percutaneous coronary intervention

Potential trial participants will receive written information of the study, and they will receive oral information by medical doctors participating in the study. Informed consent shall be obtained by a Good Clinical Practice-qualified medical doctor or research nurse participating in the study. After providing written informed consent, patients who fulfill inclusion criteria and with no exclusion criteria will be randomized according to a computer-generated random-number sequence. Block randomization is performed in a 1:1:1:1 fashion stratified by study center (Fig. 1). The study will be using SMART-TRIAL® (MEDEI ApS, Aalborg, Denmark), a combined password-protected web-based randomization module and eCRF, in each participating hospital, in order to keep the data management and the statistician blind against the study condition as long as the data bank is open. The randomization list remains with SMART-TRIAL for the whole duration of the study. Thus, randomization will be conducted without any influence of the principal investigators, raters or therapists. Patients, investigators, and all medical staff will be blinded to allocation.

**Fig. 2:**
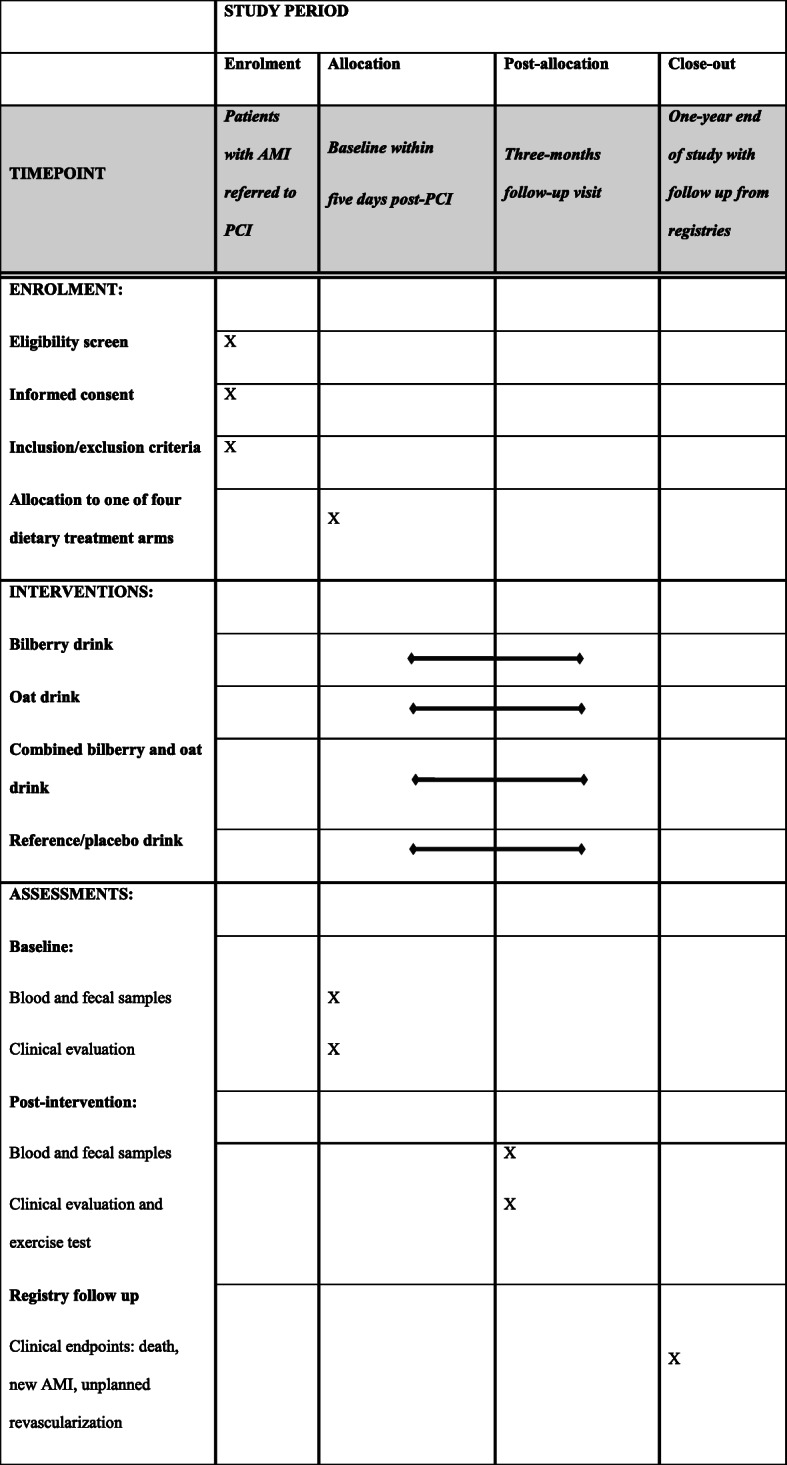
SPIRIT figure of the trial procedure including enrolment, data collection, intervention, and assessments

All randomized patients will receive standard medical and interventional treatment for AMI, including atorvastatin at a daily dose of 80 mg. The regimen for enrolment, data collection, interventions, and assessments of the trial is further shown in Fig. 2.

### Dietary intervention

Subjects will be randomized to one of the four dietary intervention arms initiated within 5 days post-PCI and continued for 3 months. After randomization, subjects will receive beverages with bilberry powder (active), beverages with high-fiber bioprocessed oat bran (active), beverages with bilberry and oats combined (active), or reference beverages containing no active bilberry or active oats, for consumption twice daily (160 kcal/day).

The beverages will be balanced to ensure isocaloric conditions. Intake twice a day is considered appropriate given the relatively short elimination half-life of anthocyanins which are believed to be important for the hypothesized effect from bilberries. The study products are under development by food scientists at Glucanova (Lund, Sweden) in collaboration with Chalmers University of Technology, Gothenburg, Sweden. Dried bilberry powder is provided by Immun (Skellefteå, Sweden). The oat bran will be bioprocessed to enable production of a palatable oat bran drink. The daily dose of oat bran will be 50 g, providing approximately 9.5 g dietary fiber of which 5 g beta-glucans, as well as abundant potentially bioactive phytochemicals, such as the oat-specific polyphenolic avenanthramides. The daily dose of the active bilberry product will contain 40 g dried bilberry powder, equal to approximately 480 g of fresh berries. Bilberry powder contains multiple anthocyanins and other polyphenols [27], the levels of the specific batches used in the present study will be analyzed in the intervention products and reported at a later date. The quantity of bioprocessed oat bran and bilberry powder in the combined study product will be 50% that of the separate supplements. The four study products are designed with similar texture and to look and taste as if they contain both bilberries and liquid oats, to enable double blinding. The reference product is based on starch, collagen, corn oil, sucrose, and citric acid. Small amounts of starch, collagen and corn oil were also added to the dried bilberry drink and the bilberry + oats drink to ensure isocaloric conditions as well as similar nutrient profiles and sensory properties (Table 3). Subjects will be instructed not to consume foods containing bilberry other than the intervention products, and no more than 15 g of oatmeal, or the equivalent, per day, during the intervention. Dietary advice according to routine treatment after AMI will be given to all study subjects.

**Table 3:** Nutritional content of study products

Nutrition (g/100g)	Dried bilberry drink	Bioprocessed oat bran drink	Bilberry + oat bran drink	Reference drink
Energy (kcal/100g)	32.1	32.7	32.0	32.3
Fat	0.6	0.9	0.9	0.7
- Of which saturated fat	0.1	0.2	0.1	0.1
Protein	0.9	2.0	1.4	1.3
Carbohydrate	4.8	3.3	3.7	5.3
- Of which sugars	3.0	2.0	2.5	2.2
Dietary fiber	1.8	1.9	1.9	0.0
- Of which β-glucan	0.0	1.0	0.5	0.0
Salt	0.06	0.05	0.00	0.02

### Examinations and blood tests

Subjects will be assessed at baseline, as inpatients, and at 3-month follow-up as outpatients. Examinations and samples will be collected as follows.

#### Blood and fecal samples

Venous blood samples will be collected at baseline and during intervention week 12. The blood samples will be centrifuged immediately and stored at – 80 °C pending further analyses. Fasting lipid profile [low-density lipoprotein cholesterol (LDL-C); total cholesterol (TC); high-density lipoprotein cholesterol (HDL-C); triglycerides (TG); small dense low-density lipoprotein (sdLDL); oxidized LDL; apolipoprotein A (apo A); apolipoprotein B (apo B); lipoprotein(a) (lp[a]); inflammatory markers high-sensitivity C-reactive protein (hs-CRP) and interleukin 6 (IL-6); fasting glucose; insulin; creatinine; cystatin C, C-peptide; glycosylated hemoglobin (HbA1c); and the heart function markers troponin-I and N-terminal pro b-type natriuretic peptide (NT-proBNP)] will be analyzed.

Analysis of blood samples will be conducted to assess diet compliance based on the presumption that the concentrations of phenolic acid derivatives will be higher in the bilberry group and avenanthramides and/or avenocosides higher in the oat and combined bilberry/oat groups compared to the reference group [27]. Phenolic acid derivative components associated with bilberry consumption [28] and avenanthramides associated with oats will be analyzed by a validated method combining liquid chromatography with mass spectrometry [29, 30].

Fecal samples will be collected at baseline and during intervention week 12. All samples will be processed and stored for metabolomics and microbiome analyses using a fecal collection kit. For metabolomics and microbiome testing, plasma and fecal samples will be collected pre- and post-intervention and stored for untargeted metabolomics and microbiota analysis at Chalmers University in Gothenburg, Sweden. Plasma samples will be processed and analyzed in four modes (reverse phase/HILIC chromatography in positive/negative ionization modes, respectively) on an LC-QTOF-MS instrument according to an established protocol [31, 32]. This will ensure the most comprehensive collection of endogenous and exposome-related metabolites. Gut microbiome will be analyzed by sequencing 16S rRNA amplicons using Illumina MiSeq. Data analysis strategies such as partial least squares (PLS) and random forest will be used to identify metabolites and microbiota that differ with treatment, as well as to detect metabolite and bacterial composition that may be related to high versus low responders to LDL-lowering treatment [33]. All analyses will be performed blinded, without knowledge of clinical diagnosis or randomization.

#### Transthoracic echocardiography

Baseline left ventricular systolic function, expressed as global ejection fraction in percent according to the biplane Simpson method, will be evaluated by echocardiography by the discharging physician. The procedure will be repeated after 3 months by an experienced echocardiography technician blinded to results of the initial examinations. The physician and the technician will be blinded to the intervention group.

#### Heart rate and blood pressure

Heart rate and blood pressure will be measured following a 15-min rest by a digital automatic sphygmomanometer (Omron m6 ac; Omron Healthcare Co, Ltd, Kyoto, Japan) at baseline and at the conclusion of the 3-month intervention. The mean of two successive measurements on each arm will be used.

#### Exercise capacity

For safety reasons, baseline recordings of exercise capacity and muscle endurance will not be performed following the acute event, and absolute values of change in exercise capacity after intervention can therefore not be estimated. The following assessments are being used in standard clinical care for all AMI patients in Sweden and will be conducted at the 3-month follow-up.

#### Symptom-limited bicycle ergometer exercise test

The test-retest reliability of the symptom-limited bicycle ergometer exercise test in patients with AMI included in the SWEDEHEART registry is excellent [34]. This submaximal exercise test will be performed on a bicycle ergometer (Monark ProVO2, Monark, Varberg, Sweden) according to a WHO protocol [35] and will be supervised by qualified physical therapists blinded to the subject intervention group. At rest, while sitting on the bicycle, subjects will be informed of the test protocol and how to rate their perceived exertion level according to the Borg RPE-scale [36] and dyspnea or chest pain according to Borg’s Category Ratio Scale (CR-10) [37]. Heart rate will be registered at rest with a wireless heart rate sensor. Systolic and diastolic blood pressure will be measured in both arms and reported for the arm with the highest value. Initial starting load, 25 W or 50 W, is based on the subject’s exertion history, with an increased in workload of 25 W every 4.5 min [38]. At 2 and 4 min of each workload, heart rate and rating of Borg scales will be registered. At 3 min, systolic blood pressure will be measured. The exercise test will be discontinued upon reaching either 17 on Borg’s RPE-scale or 7 on Borg’s CR-10 scale. Other criteria for discontinuing the test are chest pain, drop in blood pressure, failure to increase heart rate, and dizziness or other discomfort. Exercise duration of the final increment will be noted. If a subject does not complete the full 4.5 min of the final increment, a corrected maximum workload will be calculated using Strandell’s formula [39]: (submaximum workload) + (25 × *n*/4.5), where submaximal workload is the watt level prior to the termination step, and *n* is the number of minutes completed at the watt level of the final increment of exercise. Maximum oxygen uptake (VO_2_max) at highest achieved workload will be estimated with the Ekblom-Bak test (www.gih.se/ekblombaktest).

#### The dynamic unilateral heel-lift and unilateral shoulder-flexion tests

These tests are designed to evaluate muscle endurance and are commonly used in exercise-based cardiac rehabilitation settings and have excellent reliability for patients with CVD [34, 40].

#### Physical activity scales

The Frändin/Grimby activity scale and the Haskell physical activity scale will be used to subjectively measure physical activity level at baseline and follow-up [41, 42].

#### Food frequency questionnaire

All patients will complete a 132-item food frequency questionnaire with questions related to daily food intake and consumption of bilberries and oats before the study [43]. Before and after the intervention, a full 3-day diet record will be performed.

#### Register follow-up

At 1-year following randomization, we will exploratively assess clinical endpoints in all randomized patients using data from the Swedish Coronary Angiography and Angioplasty Registry (SCAAR) with respect to death, new AMI, and new unplanned revascularization.

### Sample size calculation, data management, and statistical analysis

To the best of our knowledge, no previous intervention study of bilberry/oats with LDL cholesterol as an endpoint in AMI patients has been conducted. Required sample size is calculated on the basis of bilberry-specific interventions from four smaller randomized studies in at-risk populations [18], from our pilot study [27], and from the IMPROVE-IT trial [44], which found a clinically relevant LDL cholesterol-lowering effect of − 0.4 mmol/L when adding treatment with a non-statin agent to standard statin lipid-lowering therapy after AMI. We calculated that 189 subjects would be needed in each study arm to provide 90% statistical power to detect a 0.4 mmol/L or greater reduction in LDL cholesterol in the bilberry or the oat treatment group compared to the reference, with a standard deviation of 1.1 mmol/L and a Bonferroni corrected overall 5% two-sided significance (Statistical Solutions Ltd, Cork, Ireland). The correction is due to two primary hypotheses, (1) bilberry group vs. reference and (2) oat group vs. reference. The same number of participants will be enrolled in the combined bilberry and oat treatment group, for a total of 756 patients in the study. In order to allow for an anticipated dropout rate of ~ 15%, we plan to include 900 patients, 225 in each study arm.

Data will be transferred in coded form from the participating centers to Örebro University Hospital, where data management and statistical analyses will be performed. All patients will be assigned an identifying number, recorded in the eCRF together with collected data. A code list connected to patient study numbers with individual Swedish resident personal identification numbers will be kept separately, and secured at the participating clinical centers. At completion of collection, a database with data from the eCRF and national registers will be established. The results will be analyzed according to the intention-to-treat principle; that is, subjects randomized to a given group will be followed and assessed without regard to the diet intervention eventually received. A subject can withdraw from the study at any time, if it is the wish of the subject, or if it is medically indicated, as judged by the investigator. Data collected up to the end of follow-up will be used in the final analysis of the study. If a subject wants to discontinue the study participation, data collected until that time point will be analyzed in the study. For the primary outcome, on the original scale or log scale, as appropriate, linear regression with pre-intervention to post-intervention change as outcome, adjusted for the baseline values, will be conducted. In cases of missing outcome data, multiple imputation (MI) will be used. Ordinal variables will be assessed with a *χ*^2^ test for trend or the Mann-Whitney *U* test, and Pearson’s *χ*^2^ test or Fisher’s exact test, as appropriate, will be used to evaluate differences among proportions.

To assess compliance, we will compare polyphenolic metabolites associated with bilberry and oat intake in blood at baseline and at 3 months. For values that fall below the limits of detection, an estimated concentration of 50% of the detection limit will be used. Explorative analyses of metabolome and microbiota (omics) data, will be conducted at the Department of Biology and Biological Engineering at Chalmers University of Technology. Instrumental analyses of microbiome and untargeted metabolomics will generate raw data in the range of terabytes. OMICs data will be analyzed at a server dedicated for molecular studies linked to sensitive personal data at The Swedish National Infrastructure for Computing (SNIC) using primarily multivariate strategies, incorporating unsupervised principal components analysis–based strategies and supervised PLS and random forest in-house strategies developed at Chalmers University of Technology [45].

### Administration of the trial

The steering committee, consisting of primary investigator Cecilia Bergh from the Clinical Epidemiology and Biostatistics Unit, Örebro University Hospital and sponsor Ole Fröbert of the Department of Cardiology at Örebro University Hospital, and Rikard Landberg from the Department of Biology and Biological Engineering, Food and Nutrition Science at Chalmers University of Technology and David Erlinge of the department of Cardiology at Lund University Hospital, is responsible for planning and performance of the study. At the time of protocol submission, inclusion is planned at six national sites: Sahlgrenska University Hospital Gothenburg, Umeå University Hospital, Lund University Hospital, Västerås general hospital, Karlstad general hospital, and Örebro University Hospital, all with local site investigators. Newsletters including center recruitment status and contact with local principal investigators will ensure strategies for achieving adequate participant enrolment to reach target sample size. Further sites may be added during the study period to ensure inclusion of all 900 study subjects.

### Study monitoring and data safety monitoring

The study will be monitored using SMART-TRIAL. Before beginning the clinical trial, all centers will have a web-based meeting with presentation providing a description of the study, study procedures, and documentation. During the study period, monitors will have regular telephone contact with the participating departments to ensure the trial is conducted in compliance with the protocol and applicable regulatory requirements. Monitoring will be conducted according to risk-based monitoring and a study-specific plan by personnel not otherwise involved in the study. An independent Data Monitoring Committee will not be used as this is considered a low-risk intervention.

Adherence to protocol will be continuously monitored and the responsible centers notified of violations. Based on previous research and our pilot study, we have no reason to expect negative side effects or interactions with bilberries or oats. Therefore, an interim safety analysis will not be conducted. Any side effects of the bilberry, oat or reference/placebo products will be registered according to 7b World Allergy Organization Subcutaneous Immunotherapy Systemic Reaction Grading System embedded in the eCRF.

## Discussion

In the BIOAMI study, we will investigate the potential cardiometabolic benefits of supplementation of standard post-AMI treatment with daily bilberry and/or oat intake. We will assess whether beverages with dried bilberry and/or bioprocessed oat bran lower lipid levels, reduce inflammation, and/or improve exercise capacity. Given the significant ongoing CVD burden in patients after AMI, there is a need for novel evidence-based strategies for safe secondary prevention. Diet alteration is a generally safe intervention, and international guidelines recommend diets high in fruits, vegetables, and whole grains [10]. The source of the cardioprotective effects of specific diets such as the Mediterranean and Nordic diet may be their high content of fruit, vegetable, and whole grains — foods rich in bioactive compounds and dietary fiber. Robust scientific evidence for clinical effects of specific foods is warranted and for the role of bioactive compounds that may mediate the effect. Bilberries and oats both contain phenolic compounds with potential lipid-lowering and anti-inflammatory effects [17, 23, 46]. The primary cholesterol-lowering effect of oats has been attributed to the viscous soluble fiber beta-glucans, and a cause-effect relationship has been established [16]. Other potential CVD prevention properties of oats may involve anti-inflammatory and antioxidant action as well as maintenance of endothelial function [47]. This has been suggested by in vitro and animal experiments with oat bran [48] as well as isolated oat-specific polyphenols (avenanthramides) [49–51].

Polyphenols from bilberries may affect exercise tolerance [25, 26], and, in prospective studies, a high intake of anthocyanins has been inversely associated with risk of AMI [52, 53].

Some previous trials conducted in at-risk populations have provided evidence of improved cardiovascular function following intervention with a Nordic diet that included bilberries and oats [11–13] and from daily bilberry consumption [54]. However, the potential benefit of bilberry and oat intake in patients with manifest CVD needs to be clarified in large clinical trials [15].

In contrast to previous smaller studies in at-risk populations, the proposed study is powered to evaluate clinically relevant effects on LDL cholesterol in patients following overt AMI. In addition, we will use untargeted metabolomics to detect potential biomarkers of responders and non-responders to the interventions and characterize gut microbiome/diet interactions, as well as to investigate how metabolic networks are affected by the interventions.

All AMI patients in our pilot study received statin therapy. Bilberry administration was initiated within 72 h of PCI during a vulnerable and critical clinical phase [55, 56]. Despite statin treatment, we found significant correlation of LDL cholesterol and oxidized LDL with blood levels of anthocyanins after an 8-week intervention, indicating a possible dose-response relationship of anthocyanins in bilberries with cholesterol levels. The lipid-lowering effect of bilberry could be partly explained by the high anthocyanin content [57]. Oxidized LDL is associated with all stages of atherosclerosis and with co-morbidities linked to CVD, such as diabetes mellitus, hypertension, and obesity [58]. We speculate that both bilberries and oats could play a role in supplementing medical therapy, but probably through different mechanisms, showing potential for cumulative or synergistic beneficial effects. Both bilberries and oats are natural foods that have shown potential for CVD prevention when provided separately, but their combined effects have not been studied in clinical trials nor have effects of bilberries or oats been studied in large populations with overt disease, as in this trial with 900 AMI patients. A synergistic effect of oats and bilberries could suggest development of novel cholesterol-lowering functional foods as a potential cost-effective and safe dietary strategy for reduction of cardiac risk after AMI.

In designing the intervention drinks, we faced a challenge regarding calorie intake. In order to balance the four intervention drinks calorie-wise and to avoid high contents of sucrose and starch content in the reference product, we designed the combined bilberry/oat drink to contain half the quantity of bilberry and oats in the separate drinks. Our study therefore does not fulfill 2 × 2 factorial design criteria, and this limits our ability to investigate possible interactions between bilberries and oats without making assumptions about the linear dose-response relationship.

Potential limitations also include non-adherence to the dietary regimen. However, the intervention products, developed by food scientists representing both the academic and food science fields (Chalmers University of Technology, Gothenburg, Sweden, and Glucanova, Lund, Sweden, respectively), are shelf-stable ready-to-use beverages with acceptable taste and freshness, containing natural ingredients. In order to assess compliance, we will compare polyphenolic metabolites in blood associated with bilberry and oat intake at baseline and at 3 months. Compliance with the intervention product intake will also be calculated from returned empty packages and unused drinks.

For safety reasons, baseline recordings of clinical exercise and muscle tests will not be conducted; the absolute values for changes in exercise capacity after AMI can therefore not be estimated. However, subjectively assessed physical activity will be evaluated at baseline and at 3 months using validated questionnaires. Our study is not powered to assess hard clinical endpoints such as death, new AMI events, or new unplanned revascularizations, but we will follow-up such events reported in registries at 1 year following randomization, as an exploratory analysis.

## Current trial status

The trial was registered on ClinicalTrials.gov on August 8, 2018 (ID NCT03620266). Enrolment will begin in September 2021 and is expected to continue for 2 years. All study sites are prepared to start inclusion of patients in 2021, with an amendment from the Ethical Review Board approved for inclusion of Sahlgrenska University Hospital Gothenburg and Karlstad general hospital. The final results of the primary endpoint are projected for August 2023. An open-label pilot study with 50 patients was conducted in 2014/2015 and reported in 2018 [27], and it resulted in some adjustments in the study protocol and design of the study with inclusion of oats and two more study arms, and development of the pilot product (dried bilberry powder), a liquid oat product, their combination, and a reference/placebo product for use in the intervention. Developed study products were of importance for achieving high compliance to the dietary regimen. The current protocol version is 2.0 and dated December 20, 2019.

## Supplementary Information

**Additional file 1.** Trial Registration Data Set according to WHO.

## Data Availability

Data cannot be made freely available as they are subject to privacy in accordance with the Swedish Public Access to Information and Secrecy Act but can be provided to researchers upon request, subject to a review of privacy. Requests for data can be sent to the corresponding author.

## Abbreviations

AMI: Acute myocardial infarction
apo A: Apolipoprotein A
apo B: Apolipoprotein B
CVD: Cardiovascular disease
HbA1c: Glycosylated hemoglobin
HDL: High-density lipoprotein
hs-CRP: High-sensitivity C-reactive protein
IL-6: Interleukin 6
LDL: Low-density lipoprotein
Lp(a): Lipoprotein A
NSTEMI: Non-ST-segment elevation myocardial infarction
NT-proBNP: ProBrain natriuretisk peptid
PCI: Percutaneous coronary intervention
sdLDL: Small dense low-density lipoprotein
STEMI: ST-segment elevation myocardial infarction
TC: Total cholesterol
TGA: Triglycerides
