# Supplementary material for: Effects of Bilberry and Oat intake on lipids, inflammation and exercise capacity after Acute Myocardial Infarction (BIOAMI): study protocol for a randomized, double-blind, placebo-controlled trial
Source: Trials. 2021 May 10;22:338. doi: 10.1186/s13063-021-05287-5 (PMC8112057; doi:10.1186/s13063-021-05287-5)
Supplement: Supplementary file 1 — Additional file 1. Trial Registration Data Set according to WHO. [file 13063_2021_5287_MOESM1_ESM.docx]

Additional File 2. Trial Registration Data Set according to WHO

| **Data category** | **Information** |
| --- | --- |
| Trial identifying number | ClinicalTrials.gov, ID NCT03620266 |
| Date of registration | August 8, 2018 |
| Sources of monitary or material support | Örebro University Hospital, Region Örebro Lan |
| Primary sponsor | Ole Fröbert, MD, PhD, Region Örebro Lan |
| Contact for public and scientific queries | Ole.frobert@regionorebrolan.se |
| Public and scientific title | Effects of Bilberry and Oat intake on lipids, inflammation and exercise capacity after Acute Myocardial Infarction (BIOAMI): a randomized, double-blind, placebo-controlled trial |
| Country of recruitment | Sweden |
| Health conditions studied | Lipid profile, inflammation and exercise capacity after acute myocardial infarction. |
| Interventions | Dietary supplements with drinks supplemented with either dried bilberry, liquid oats, a combination of bilberry and oats or a reference/placebo drink. |
| Key inclusion criteria | STEMI or NSTEMI, completed coronary angiography/PCI, male and female subjects ≥18 years, allocated to atorvastatin at a daily dose of 80 mg. |
| Key exclusion criteria | Emergency coronary artery bypass grafting, <18 years of age, LDL cholesterol <2.0 mmol/L, daily intake or the intent to initiate daily intake of bilberry in any form or daily intake of >15 g of oatmeal or equivalent. |
| Study type | Interventional  Allocation: randomized, Intervention model: parallel assignment, Masking: Quadruple (Participant, Care Provider, Investigator, Outcomes Assessor), Four treatment arms.  The primary purpose is treatment and secondary prevention after acute myocardial infarction. |
| Date of first enrolment | January 15, 2021 |
| Target sample size | 900 |
| Recruitment status | Not yet recruiting |
| Primary outcomes | LDL-C cholesterol change between the treatment groups after three months of diet supplementation with bilberry and/or oats. |
| Key secondary outcomes | Exercise capacity, plasma concentrations of biochemical markers of inflammation, metabolomics and gut microbiota composition after three months. |
